# Clinical Assessment of Moringa oleifera as a Natural Crosslinker for Enhanced Dentin Bond Durability: A Randomized Controlled Trial

**DOI:** 10.7759/cureus.46304

**Published:** 2023-10-01

**Authors:** Lavanya Anumula, Sindhu Ramesh, Suneel Kumar Chinni, Prasanth Punamalli, Venkata Suneel Kumar Kolaparthi

**Affiliations:** 1 Conservative Dentistry and Endodontics, Narayana Dental College and Hospital, Nellore, IND; 2 Conservative Dentistry and Endodontics, Saveetha Dental College and Hospitals, Saveetha Institute of Medical and Technical Sciences, Saveetha University, Chennai, IND; 3 Public Health Dentistry, Narayana Dental College and Hospital, Nellore, IND; 4 Oral Medicine and Radiology, Narayana Dental College and Hospital, Nellore, IND

**Keywords:** collagen, antioxidants, flavonoids, adhesives, matrix metalloproteinases

## Abstract

Background: Dentin biomodification is a biomimetic approach that strengthens the collagen network, making it less susceptible to enzymatic degradation and improving the durability of bonded restorative materials, using collagen crosslinkers.

Objective: This study aimed to assess the effectiveness of Moringa oleifera as a natural crosslinker in improving the clinical success of resin-dentin restorations.

Method: A double-blind, controlled, randomized clinical trial was conducted in accordance with Consolidated Standards of Reporting Trials (CONSORT) guidelines, with 50 adult participants with initial carious lesions (ICDAS 4 and 5) enrolled. Participants were randomly assigned to either the experimental group (which received Moringa oleifera as a pretreatment liner) or the control group (standard restorative procedures without a liner). Functional and biological outcomes were assessed at baseline, six months, and 12 months using the FDI criteria. Statistical analysis included Fisher's exact test, Wilcoxon sign rank test, and Mann-Whitney U test.

Results: Both groups exhibited excellent functional properties and marginal adaptation at baseline and six months. At the 12-month mark, the test group displayed clinically better functional properties (97.9%, n=47) compared to the control group (95.8%, n=46), but there was no significant difference (p-value>0.05). Marginal gaps were observed in both groups at six and 12 months (8.3%, n=4), with no significant inter-group variation (p-value>0.05). Radiographic examination showed a harmonious restoration-to-tooth transition. Patient satisfaction remained high, with the test group 4.2% (n=2) and control 2.1% (n=1) reporting minor issues at 12 months, though not statistically significant (p-value>0.05). Postoperative sensitivity was minimal, and tooth integrity was well-preserved.

Conclusion: Moringa oleifera, as a pretreatment liner, showed promise in enhancing the clinical success of resin-dentin restorations. Despite minor reported issues, the groups had no statistically significant differences regarding functional and biological outcomes.

## Introduction

In contemporary restorative dentistry, ensuring the durability of the resin dentin bond is just as crucial as restoring function and aesthetics. Despite advancements in dental resin composite restorations, long-term success remains challenging due to microleakage, secondary caries, and marginal degradation [1]. One of the primary reasons for this is the vulnerability of adhesive interfaces to degradation over time, particularly due to the presence of endogenous enzymes such as matrix metalloproteinases (MMPs) found in the dentin matrix [2].

These MMPs can break down collagen fibrils in the dentin, leading to a deteriorating bond, microleakage, and eventual restoration failure. In order to enhance the stability of resin-dentin interfaces, several methods have been developed to inhibit collagen degradation and improve the mechanical properties of the hybrid layer. One such method is dentin biomodification, which is a novel and biomimetic approach [3]. Biomodification involves modifying existing hard tissue structures, such as tooth dentin, to strengthen the collagen network, making it less susceptible to enzymatic degradation and controlling biodegradation rates of extracellular matrix components [4]. This results in a significant improvement in the overall bond strength and durability of restorative materials [5]. The crosslinking agents improve the covalent intermolecular crosslinks and also possess an inhibitory effect on endogenous proteases such as MMPs, thus preventing the disruption of the hybrid layer [6].

Although the use of crosslinkers as a pretreatment liner has been explored to enhance the longevity of resin-dentin bonds, there are concerns regarding the potential toxicity and biocompatibility of synthetic crosslinkers [7]. Natural alternatives for crosslinking have been gaining attention due to their biocompatibility, potential for reduced toxicity, and sustainability [8]. One such natural substance that has shown promise is Moringa oleifera, commonly known as the drumstick tree or horseradish tree. It has been investigated for its phenolic and flavonoid content and tested for its effect on collagen degradation (telopeptide estimation) and resin dentin bond durability (unpublished data). M. oleifera also contains bioactive compounds that have been shown to have antioxidant, anti-inflammatory, and antibacterial properties, making it a promising natural crosslinker [9].

Despite the growing interest in natural crosslinkers, there is still a lack of studies evaluating their effectiveness in enhancing resin-dentin bonds in long-term clinical settings. Currently, there is a notable absence of research regarding the prolonged utilization of M. oleifera as a pretreatment liner and its subsequent assessment using standardized clinical criteria.

The purpose of this study was to investigate whether using M. oleifera, a natural crosslinker, as a pretreatment liner could improve the clinical success of the restoration. The research was conducted using a randomized clinical trial design that followed the guidelines of the FDI World Dental Federation. The null hypothesis was that there was no difference in the clinical performance of composite restoration in groups with or without Moringa extract as a pretreatment liner.

## Materials and methods

Trial design: The study design adhered to the guidelines in the Consolidated Standards of Reporting Trials (CONSORT) statement [10]. It was a controlled, double-blind, randomized clinical trial, following authorization from the institutional review board (IEC/NDCH, 2019/P-24). The trial's registration took place in the Clinical Trial Registry - India, with registration number CTRI/2021/04/033069. The study was conducted at the Department of Conservative Dentistry and Endodontics from June 2021 to November 2021. Before their involvement, all participants received comprehensive information about the study's objectives and aims, though they were unaware of which specific treatments were administered to individual teeth.

**Figure 1:**
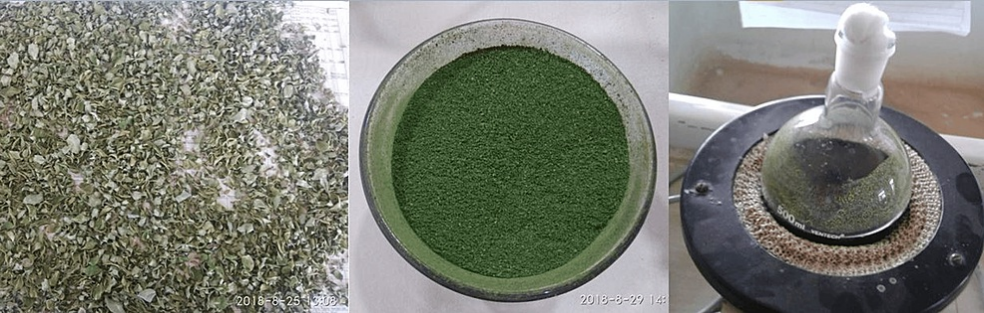
Preparation of Moringa extract Shade-dried leaves of Moringa, which were powdered and subjected to hydroalcoholic extraction by cold percolation method.

Preparation of Moringa leaf extract: The shade-dried leaves of M. oleifera were powdered and subjected to hydroalcoholic extraction by cold percolation method. About 10 g of the shade-dried and coarsely powdered plant material was soaked in 100 ml of 70% ethanol solution for 24 h (Figure 1). The resulting extract was then filtered through Whatman No. 1 filter paper, and the filter cake was collected. The cake was dried at 37 °C, and the resulting extract was stored at 4 °C. To achieve the desired concentration of 5%, dimethyl sulfoxide (DMSO) was employed. This resulted in the 5% Moringa solution. 

Participants: 50 adult participants, between 18 and 65 years presenting with at least two carious lesions requiring restoration, were recruited for the study.

Inclusion and exclusion criteria: Participants were screened for eligibility based on the following inclusion criteria. Presence of two posterior carious lesions with an initial carious lesions (ICDAS) scoring of 4 or 5 [11], good general health without any systematic disorders that could interfere with the study, willingness to comply with the study protocol and informed consent.

Participants with known allergies to Moringa or any dental restorative material used in the study, pregnant or lactating mothers, and multiple posterior carious lesions requiring extensive restorations were excluded.

Sample size: The sample size of 50 participants was determined based on the effect size of 0.80, an α value of 0.05, 80% power, and an anticipated dropout rate. It was determined that the sample size was adequate for detecting notable distinctions between the material groups in comparable intra-individual comparison designs.

Randomization and blinding: To ensure unbiased results, a split-mouth design was employed, dividing the oral cavity into left and right halves, with all included patients. This design allowed for assessing the impact of the experimental procedure on one side of the mouth while comparing it with the other. By using each participant as their own control, variability was reduced, enhancing the study's accuracy, all while minimizing the need for a large number of participants.

In this trial, the split-mouth design was applied by treating teeth on opposite sides of the oral cavity differently. One tooth received a 5% M. oleifera extract as a pretreatment liner (experimental group), while the other tooth underwent the standard restorative procedure without a liner (control group). A block of four numbers was used from a random table to select which tooth received the treatment. If the number was odd, the moringa extract was applied to the first tooth, and if it was even, the procedure proceeded without a liner. The initial tooth was selected based on its order in the FDI notation.

The randomization and grouping of the subjects are presented in Figure 1 (CONSORT flow diagram). The participants and investigators assessing the outcomes were blinded to the group allocation.

**Figure 2:**
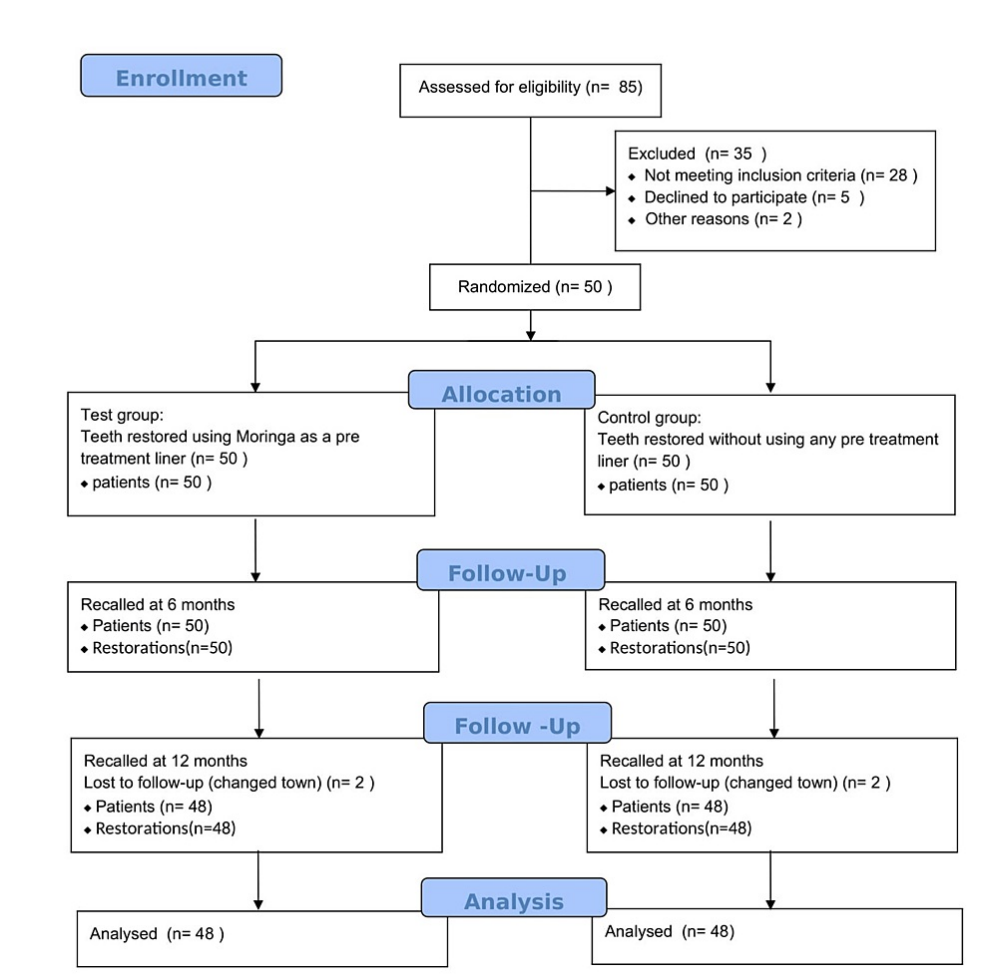
CONSORT flow diagram The figure depicts the flow of participants through the trial, i.e., enrollment, allocation, follow-up, and analysis.

**Figure 3:**
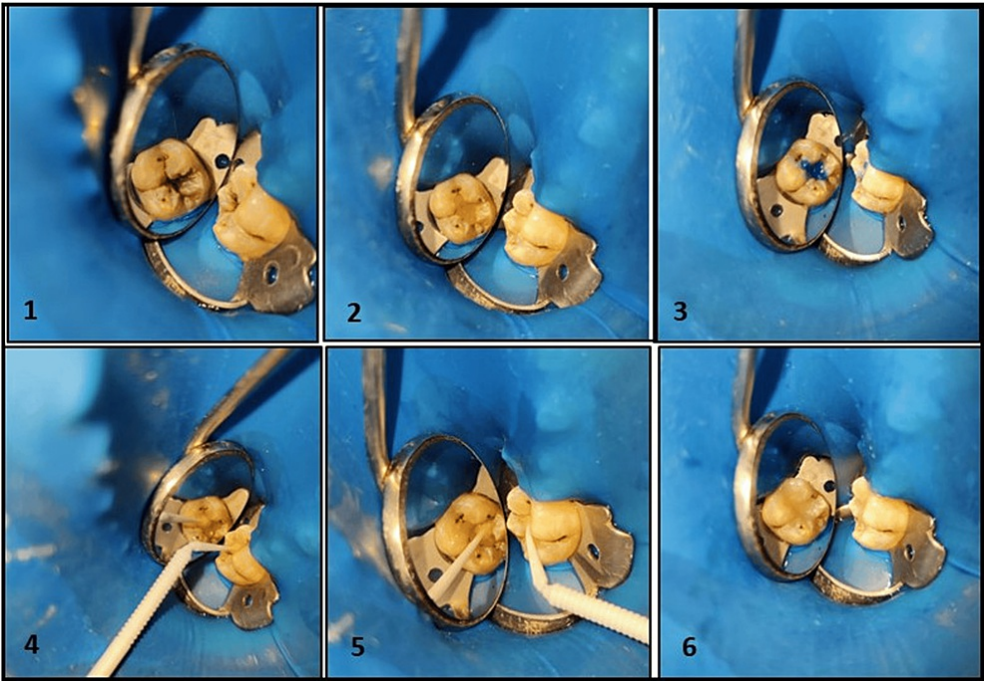
Clinical steps for placing composite restoration with the Moringa pretreatment liner 1. Carious lesion (ICDAS 5), 2. Tooth preparation, 3. Total etching (followed by rinsing and drying), 4. Application of the Moringa pretreatment liner for a minute and dried, 5. Application of a bonding agent (followed by curing), 6. Composite restoration

Clinical procedure: Treatment was performed according to a predetermined procedure, which included prophylaxis and shade selection with the VITA Classical shade guide. Participants who met the eligibility criteria received restorations bonded with or without a pretreatment liner. Class 1 tooth preparations were prepared using high-speed air rotor 245 burs under rubber dam isolation. The carious lesion was eliminated. After etching the tooth in one quadrant, 5% moringa extract solution, as a pre-treatment liner, was applied to all the cavity walls and floor using a micro applicator for a duration of approximately one minute (Figure 3). Thereafter, the liner was allowed to dry for a period of one minute.

**Figure 4:**
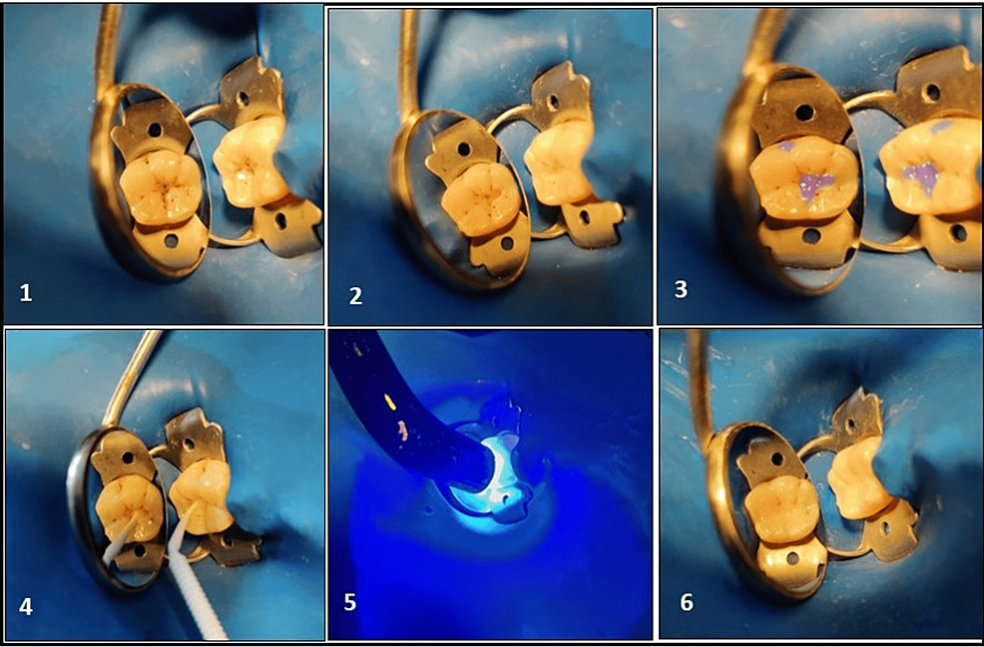
Clinical steps for placing composite restoration without the pre-treatment liner (control group) 1. Carious lesion (ICDAS 4), 2. Tooth preparation 3. Total etching (followed by rinsing and drying), 4. Application of a bonding agent, 5. Light curing of the bonding agent, 6. Composite restoration

Control group (Figure 4): After the caries removal, the participants undergo the standard dental restorative procedure without applying any pretreatment liner.

This was followed by applying a universal bonding agent (Tetric N-Bond, Ivoclar Vivadent, Liechtenstein) and resin composite restoration, in both groups (Tetric N-Ceram, Ivoclar Vivadent, Liechtenstein). After the restorative procedure, the final contour was achieved using a fine diamond rotary instrument. The restorations were polished with rubber points (Astropol F, Ivoclar Vivadent, Liechtenstein).

Evaluation: The restoration was evaluated using the FDI criteria [12] at baseline, six months, and 12 months by two blinded, calibrated clinicians not involved with the treatment procedure.

Out of the 16 FDI properties, eight properties that were relevant to this study were selected. The properties were fracture and retention, marginal adaptation, patients' view, radiographic examination, postoperative sensitivity, recurrence of caries, tooth integrity, and oral and general health of the patient [12] (Table 1).

**Table 1:** FDI evaluation criteria of functional and biological properties The selected functional and biological properties [12] that were evaluated in the follow-up intervals for a Class I composite restoration

Properties		Criteria
Fracture of material and retention	1	No fractures /cracks
	2	Small hairline crack
	3	Two or more or larger hairline cracks and/or material chip fractures not affecting the marginal integrity or approximal contact
	4	Material chip fractures which damage the marginal quality or approximal contacts. Bulk fractures with partial loss (less than half of the restoration)
	5	(Partial or complete) loss of restoration or multiple fractures
Marginal adaptation	1	Harmonious outline, no gaps, no white or discoloured lines
	2	Marginal gap (<150 µm), white lines, Small marginal fracture removable by polishing, Slight ditching, slight step/flashes, minor irregularities
	3	Gap < 250µm not removable, several small marginal fractures, Major irregularities, ditching or flash steps
	4	Gap > 250µm or dentine/base exposed, severe ditching or marginal fractures, larger irregularities or steps (repair necessary)
	5	Restoration (complete or partial) is loose but in situ, Generalized major gaps or irregularities
Patient's view	1	Entirely satisfied with esthetics and function
	2	Satisfied, Esthetics, Function, e.g., minor roughness
	3	Minor criticisms but no adverse clinical effects. Esthetic shortcomings. Some lack of chewing comfort. Unpleasant treatment procedure
	4	Desire for improvement, Esthetics, Function, e.g., tongue irritation, reshaping of anatomic form or refurbishing is possible
	5	Completely dissatisfied and/or adverse effects, including pain
Radiographic examination	1	No pathology, harmonious transition between restoration and tooth
	2	Acceptable material excess present, Positive/negative step present at margin <150 µm
	3	Marginal gap < 250 µm, Negative steps visible < 250 µm. No adverse effects noticed, Poor radiopacity of filling material
	4	Marginal gap >250 µm, Material excess accessible; but not removable, Negative steps >250µm and repairable
	5	Secondary caries, large gaps, large overhangs, Apical pathology. Fracture/loss of restoration or tooth
Post operative Hypersensitivity	1	No hypersensitivity, normal vitality
	2	Minor hypersensitivity for a limited period of time, normal vitality
	3	Moderate hypersensitivity, Delayed/mild sensitivity; no subjective complaints, no treatment needed
	4	Intense hypersensitivity. Delayed with minor subjective symptoms. No clinical detectable sensitivity. Intervention necessary but not replacement
	5	Intense, acute pulpitis or non-vital tooth. Endodontic treatment is necessary and restoration has to be replaced
Recurrence of caries	1	No secondary or primary caries
	2	Small and localized Demineralization, Erosion or Abfraction
	3	Larger areas of De-mineralisation, Erosion or Abrasion/abfraction, dentine not exposed. Only preventive measures necessary
	4	Caries with cavitation and suspected undermining caries, Erosion in dentine, and Abrasion/ abfraction in dentine. Localized and accessible can be repaired
	5	Deep caries or exposed dentine that is not accessible for repair of restoration
Tooth integrity	1	Complete integrity
	2	Small marginal enamel fracture (<150 µm). Hairline crack in enamel (<150 µm); Marginal enamel defect <250 µm
	3	Crack <250 µm; Enamel chipping. Multiple cracks.
	4	Major marginal enamel defects; gap > 250 µm or dentine or base exposed. Large cracks >250 µm, probe penetrates. Large enamel chipping or wall fracture
	5	Cusp or tooth fracture
Oral and general health	1	No oral or general symptoms
	2	Minor transient symptoms of short duration; local or generalized
	3	Transient symptoms, local and/or general
	4	Persisting local or general symptoms of oral contact stomatitis or lichen planus or allergic reactions. Intervention necessary but no replacement
	5	Acute/severe local and/or general symptoms

The primary outcome was the clinical success of the restoration. Clinical success was defined as the restorations being present without any complications, as Jongsma et al. [13] stated. The study's secondary outcome was based on the eight criteria mentioned above.

Statistical analysis: The data collected at baseline, six months, and 12 months of follow-up were analyzed using statistical methods. The Fisher's exact test in Statistical Product and Service Solutions (SPSS) (version 22; IBM SPSS Statistics for Windows, Armonk, NY) was used for tabulation and scoring. Intra-group evaluation between time periods was carried out using the Wilcoxen signed-rank test. Inter-group evaluation was performed with the Mann-Whitney U test.

## Results

Table 2 displays the scores obtained at baseline (one week), six months, and 12 months. Irrespective of the pretreatment liner, all restorations were rated as clinically excellent or clinically good. Out of the 50 patients, all attended the follow-up at six months, while only two were lost to follow-up at 12 months.

**Table 2:** Comparison of the clinical evaluation of functional and biological properties of restorations at baseline, six months, and 12 months Based on the criteria, all the restorations were scored either 1 or 2. P value <0.05, no significant difference between the two groups

	Test group			Control group			
	Moringa pretreatment liner n(%)			Without Pre treatment liner n(%)			Fisher's exact test
Functional & Biological Properties-FDI	Baseline	6 months	12 months	Baseline	6 months	12 months	p value
	n=50	n=50	n=48	n=50	n=50	n=48	
Fracture of material and retention							
1 Clinically excellent	50 (100)	50(100)	47 (97.9)	50 (100)	50 (100)	46 (95.8)	Baseline & 6 months - NS
2 Clinically good	0	0 (0)	1 (2.1)	0	0	2 (4.2)	12 months: 0.315 (NS)
Marginal adaptation							
1 Clinically excellent	50 (100)	47 (94)	44 (91.7)	50 (100)	45 (90)	44 (91.7)	Baseline & 12 months - NS
2 Clinically good	0	3 (6)	4 (8.3)	0	5 (10)	4 (8.3)	6 months: 0.715 (NS)
Patients' view							
1 Clinically excellent	50 (100)	50 (100)	46 (95.8)	50 (100)	50 (100)	47 (97.9)	Baseline & 6 months - NS
2 Clinically good	0	0	2 (4.2)	0	0	1 (2.1)	12 months: 0.557 (NS)
Radiographic examination							
1 Clinically excellent	50 (100)	50 (100)	48 (100)	50 (100)	50 (100)	48 (100)	Baseline, 6, & 12 months - NS
Postoperative Hypersensitivity							
1 Clinically excellent	50 (100)	50 (100)	47 (97.9)	50 (100)	50 (100)	48 (100)	Baseline & 6 months - NS
2 Clinically good	0	0	1 (2.1)	0	0	0	12 months: -0.557 (NS)
Recurrence of caries							
1 Clinically excellent	50 (100)	50 (100)	48 (100)	50 (100)	50 (100)	47 (97.9)	Baseline & 6 months - NS
2 Clinically good	0	0	0	0	0	1 (2.1)	12 months: 0.315 (NS)
Tooth integrity							
1 Clinically excellent	50 (100)	50 (100)	48 (100)	50(100)	50(100)	48 (100)	Baseline, 6, & 12 months - NS
Oral and General Health							
1 Clinically excellent	50 (100)	50 (100)	48 (100)	50 (100)	50 (100)	48 (100)	Baseline, 6, & 12 months - NS

Functional properties

Fracture and retention: Both the test group (teeth restored using Moringa as a pretreatment liner) and the control group (restored without any liner) exhibited excellent functional properties, without any fractures or cracks at baseline, as well as at six months, follow-up. At 12 months, the test group continued to display clinically excellent functional properties (97.9%, n=47) with fewer small hairline cracks than the control group (95.8%, n=46). However, the observed difference was insignificant (p-value>0.05).

Marginal adaptation: Both the test and control groups had excellent marginal adaptation at the start of the study, with no gaps or discolored lines. However, after six and 12 months, both groups had marginal gaps (8.3%, n=4). The control group had slightly more discrepancies (10%, n=5) at the six months compared to the test group (6%, n=3), but this difference was not statistically significant (p-value>0.05).

Patients’ view: The participants in both groups reported complete satisfaction with esthetics and function at baseline and six-month follow-up. However, at 12 months of follow-up, though satisfaction remained high, a small percentage of participants in both the test and control groups reported minor issues such as roughness. Specifically, 4.2% (n=2) of participants in the test group and 2.1% (n=1) in the control group reported such issues. Despite the difference in percentages, statistical analysis revealed that these differences were not significant (p-value>0.05).

Radiographic examination: Radiographically, both groups demonstrated a harmonious transition between restoration at baseline, six months, and 12 months of follow-up.

Biological properties

Postoperative sensitivity: There were no reports of hypersensitivity in either group at baseline and six months of follow-up. However, after 12 months of follow-up, one patient in the test group experienced transient hypersensitivity with normal vitality. It is worth noting that this finding was not deemed statistically significant (p-value>0.05).

Recurrence of caries: Both the control and experimental groups showed no signs of secondary or primary caries at the beginning and after six months. However, after 12 months, a single patient from the control group reported localized demineralization/erosion. The results showed no significant differences between the groups, with a p-value greater than 0.05.

Tooth integrity: Complete tooth integrity was maintained in both groups at baseline and throughout the six and 12 months of follow-up periods.

Oral and general health: No oral or general health symptoms were reported in either group throughout the study period.

Inter-group and intra-group comparisons

Both groups exhibited no significant difference in inter- and intra-group comparisons (data not shown). During the six-month study period, the control group showed slight differences when compared with the experimental group. These differences were found to be significant, with a p-value of 0.025.

## Discussion

The long-term success of composite restoration largely depends on the durability of dentin bonding. However, the stability of dentin collagen fibrils is a prerequisite for effective bonding. MMPs pose a threat to these collagen fibrils as they are susceptible to enzymatic degradation, ultimately leading to bond degradation. To improve the stability of collagen fibrils, biomodification of dentinal collagen using crosslinking agents is recommended as a prebonding strategy [14]. Several natural and synthetic materials have been used as crosslinkers to improve the durability of dental bonds.

Considering the growing interest in natural or nature-based solutions due to biosafety and clinical feasibility offered by these alternatives, many plant extracts, such as proanthocyanidin [15], epigallocatechin-3-gallate (EGCG) [16], quercetin [17], and baicalein [18], are considered potential sources of bioactive compounds with antioxidant, antimicrobial, and anti-inflammatory effects.

Among these plants, M. oleifera stands out as a candidate due to its rich content of polyphenols and flavonoids and, thus, can be a potential cross-linking agent [19]. Thus, the study aimed to evaluate the potential of M. oleifera as a natural crosslinker, contributing to enhanced adhesive properties and ultimately improving the longevity and performance of resin composite restorations. With regard to parameters, no statistically significant difference was reported in the clinical performance of composite restoration in groups with or without Moringa extract as a pretreatment liner. Therefore, the null hypothesis was accepted.

The FDI criteria provide a set of standards to assess the success of direct and indirect restoration procedures. These criteria are flexible, well-structured, and more sensitive than the modified US Public Health Service (USPHS) guidelines in detecting small differences in restorations [20]. They offer a comprehensive framework for evaluating restoration performance based on esthetic, functional, and biological parameters, with each parameter further divided into four, five, or six criteria. Each criterion is rated on a five-point scale, with scores of up to 3 indicating acceptability and 4 and 5 indicating non-acceptability. This study focused on eight out of the 16 parameters relevant to the research question [12]. According to the FDI criteria, both the groups of restorations, with and without moringa extract as a pretreatment liner, were rated clinically excellent or clinically good at baseline, six months, and 12 months.

The restorations in the test and control groups were performed in the same patients, thus removing a lot of inter-individual variability from the estimates of the treatment [21]. This ensured that both groups were subjected to similar environmental conditions and had comparable susceptibility to caries. To prevent experimental bias, a single operator conducted all the restorations. These restorations were evaluated by two unbiased and calibrated clinicians not part of the treatment process.

When assessing restorations in clinical practice, restoration fracture and retention are the two main categories to consider. These categories encompass various issues, such as cracks, chipping/delamination, bulk fractures, and incomplete or complete loss of retention [22]. However, in this study, none of these issues were present, and both groups reported clinical success as the restorations were retained, thus achieving the primary objective. Marginal adaptation was predominantly similar between the groups, with minor discrepancies in the control group not reaching statistical significance. Patient satisfaction remained high, with minor issues reported, primarily minor roughness in the test group at a 12-month follow-up, which might be attributed to various factors unrelated to the intervention. Radiographic assessments showed no signs of pathology, aligning with the absence of secondary caries. Both the groups maintained complete tooth integrity and overall general health. 

While the study's results did not demonstrate statistically significant differences between the groups, the potential benefits of using M. oleifera in enhancing resin-dentin bond durability cannot be dismissed. Additionally, the absence of significant complications or differences in biological properties, such as postoperative sensitivity, tooth integrity, and oral and general health, highlights the safety profile of M. oleifera as a potential natural crosslinker.

While our study has revealed encouraging trends, it is essential to acknowledge certain limitations. Firstly, the preparation of fresh extracts for natural crosslinkers can pose a challenge in clinical settings, and commercially obtained materials may raise concerns about their purity. These practical considerations are of utmost importance when utilizing natural crosslinkers in dental applications to ensure the efficacy and safety of the final product. Additionally, although our study did not find statistically significant differences, further investigations through multi-center trials with more extended follow-up periods are necessary to capture potential long-term effects and delayed complications that may arise over extended durations.

## Conclusions

To summarize, this research offers valuable initial insights into the potential of using M. oleifera as a natural crosslinker to strengthen the bond between resin and dentin, increasing its durability. Although the results did not show significant differences between the experimental and control groups, there were positive trends in clinical excellence, functionality, and marginal adaptation. These trends highlight the safety and potential advantages of using natural crosslinkers such as M. oleifera in restorative dentistry. Therefore, further research is encouraged through future multi-center trials with extended follow-up periods to study any long-term effects and delayed complications that may arise, advancing our understanding of the use of natural crosslinkers in dental applications.
